# *OsYSL13* Is Involved in Iron Distribution in Rice

**DOI:** 10.3390/ijms19113537

**Published:** 2018-11-09

**Authors:** Chang Zhang, Kamran Iqbal Shinwari, Le Luo, Luqing Zheng

**Affiliations:** 1 College of Life Sciences, Nanjing Agricultural University, Nanjing 210095, China; 2014216025@njau.edu.cn (C.Z.); kamishin@yahoo.com (K.I.S.); 2 College of Resources and Environmental Sciences, Nanjing Agricultural University, Nanjing 210095, China; luole@njau.edu.cn

**Keywords:** distribution, iron, *OsYSL13*, rice, transporter

## Abstract

The uptake and transport of iron (Fe) in plants are both important for plant growth and human health. However, little is known about the mechanism of Fe transport in plants, especially for crops. In the present study, the function of *yellow stripe-like 13* (*YSL13*) in rice was analyzed. *OsYSL13* was highly expressed in leaves, especially in leaf blades, whereas its expression was induced by Fe deficiency both in roots and shoots. Furthermore, the expression level of *OsYSL13* was higher in older leaves than that in younger leaves. *OsYSL13* was located in the plasma membrane. Metal measurement revealed that Fe concentrations were lower in the youngest leaf and higher in the older leaves of the *osysl13* mutant under both Fe sufficiency and deficiency conditions, compared with the wild type and two complementation lines. Moreover, the Fe concentrations in the brown rice and seeds of the *osysl13* mutant were also reduced. Opposite results were found in *OsYSL13* overexpression lines. These results suggest that *OsYSL13* is involved in Fe distribution in rice.

## 1. Introduction

Iron (Fe) is one of the essential microelements for both plants and humans. It exists in multiple redox states and has vital roles in diverse physiological processes such as the photosynthesis, respiration, chlorophyll biosynthesis, production, and scavenging of reactive oxygen species [1,2]. Plant growth will be inhibited and the yield will be reduced under Fe deficiency [1,3]. For humans, Fe deficiency is one of the most common forms of micronutrient malnutrition (MNM) in the world, and it results in around 0.8 million deaths (1.5% of the total) each year (WHO publications, http://www.who.int/nutrition/publications/micronutrients). Apart from its beneficial functions, Fe also can become toxic when present in excess [4]. In order to keep Fe at a suitable concentration in plants, multiple transporters, including the yellow stripe-like (YSL) transporter family, are involved in Fe absorption, translocation, distribution, and storage [2,5,6,7].

There are eighteen yellow stripe-like (YSL) members in rice and eight members in *Arabidopsis thaliana*, which can be divided into four sub-family groups [5]. *OsYSL2*, *9*, *15*, and *16* and *AtYSL1*, *2*, and *3* belong to Group I. They have the highest sequence similarity to *ZmYS1,* which is responsible for Fe(III) absorption in maize [8,9]. Most of them participate in Fe homeostasis for their Fe(III)-deoxymugineic acid (DMA) and/or Fe(II)-nicotianamine (NA) transport activity [10,11,12,13,14,15,16,17,18,19,20,21]. It has been reported that *OsYSL2* played a crucial role in long-distance Fe translocation and accumulation in grains [13,14]. *OsYSL9* is essential for Fe translocation from the endosperm to embryo [20]. *OsYSL15* takes part in Fe uptake [12,17]. *OsYSL16* is also involved in Fe homeostasis at the seedling stage, while the exact mechanism is unclear [15,18]. Besides, *OsYSL16* located in the phloem transports copper (Cu)-NA from the old tissues to the new developing ones [22,23]. *AtYSL1* is involved in long-distance Fe translocation and delivery to the seeds [16]. Furthermore, reduced concentrations of Fe, Cu, and zinc (Zn) in the seeds of the *atysl1atysl3* double mutant were found [10]. *AtYSL2* may also be involved in Fe homeostasis for its expression pattern and transport activity, while more physiological data are still lacking [11,19].

*OsYSL5*, *6* and *AtYSL4*, *6* belong to Group II. *OsYSL6* is a manganese (Mn)-NA transporter with an unclear subcellular localization. It was reported that growth inhibition was more severe in the *osysl6* mutant than that in the wild type under the Mn excess condition [24]. An increased Mn concentration in the leaf apoplastic solution and a decreased one in the symplastic solution were found in the *osysl6* mutant, suggesting that *OsYSL6* was responsible for the detoxification of excess Mn [24]. *AtYSL6* is located at the chloroplast envelop and takes part in Fe translocation out of the chloroplasts for the increased Fe concentration in the chloroplasts of the *atysl4atysl6* double mutant [25]. *AtYSL4* may also have a similar function to *AtYSL6* [25]. Besides, another study reported that *AtYSL4* and *AtYSL6* were located in the vacuole membranes and the internal membranes resembling endoplasmic reticulum [26]. Similarly, both of them reported that the *atysl4atysl6* double mutant was more sensitive to Fe excess, although their transport substrates were still unknown [25,26]. Group IV consists of 7 YSL members in rice, including *OsYSL1*, *3*, *4*, *7*, *8*, *17,* and *18*. Among them, *OsYSL18* was the only one gene that has been reported. OsYSL18, an Fe(III)-DMA transporter, is mainly expressed in the flower and may be involved in Fe translocation in the reproductive organs [27].

The remaining members belong to Group III. However, few studies focus on their function about micronutrient homeostasis. Among these members, *OsYSL13* was found to be induced in the roots under Fe deficiency by microarray analysis [28]. In this study, the role of *OsYSL13* in Fe homeostasis was investigated by examining its expression pattern and analyzing Fe concentrations in the T-DNA insertional mutant and overexpression lines of *OsYSL13*.

## 2. Results

### 2.1. Expression and Subcellular Localization of OsYSL13

**Figure 1:**
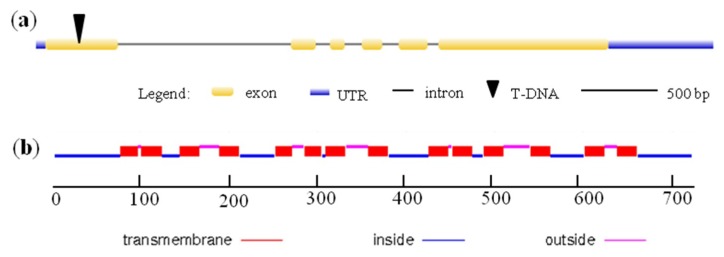
Sequence analysis of rice *yellow stripe-like 13* (*OsYSL13*) in rice. (**a**) The gene structure of *OsYSL13*. The cDNA of *OsYSL13* was cloned from the cDNA library of *Oryza sativa* cv. *Nipponbare* and its sequence were consistent with the sequence published in the GenBank/EMBL databases (accession number Os04g0524500). Exon, intron, and 5′ and 3′ untranslated region (UTR) were shown. The T-DNA was inserted into the first exon. (**b**) Predicted transmembrane domains of *OsYSL13* by TMHMM2.0. There were 14 transmembrane domains in *OsYSL13*.

*OsYSL13* consists of six exons and five introns (Figure 1a), encoding a protein of 724 amino acids. *OsYSL13* shares 51% sequence similarity to ZmYS1 and is predicted as a membrane protein with 14 transmembrane domains by SOSUI program and TMHMM2.0 (Figure 1b).

**Figure 2:**
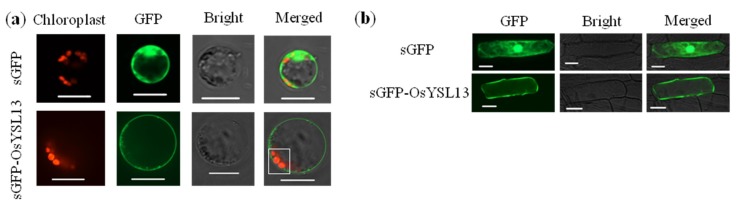
Subcellular localization of *OsYSL13*. (**a**,**b**) Subcellular localization of *OsYSL13* in the rice leaf protoplasts (**a**) and onion epidermal cells (**b**). sGFP-OsYSL13 construct was transiently transformed into the rice leaf protoplasts and onion epidermal cells. Fluorescent signals were observed 16 h after transformation. The red autofluorescence represents the location of the chloroplasts, and the green signal indicates the position of GFP or GFP-OsYSL13 fusion protein. The white box shows the relative position of GFP-OsYSL13 fusion protein and chloroplast. Scale bars = 15 and 60 μm in **a** and **b**, respectively.

In order to investigate the potential function of *OsYSL13*, subcellular localization was determined by the transient expression of the sGFP-OsYSL13 fusion protein in the rice leaf protoplasts and onion (*Allium cepa*) epidermal cells. GFP fluorescence signal was mainly observed in the plasma membrane (Figure 2a,b), which was located outside of the chloroplast (Figure 2a). The results suggested that *OsYSL13* was involved in intercellular transport but not intracellular transport of metal.

**Figure 3:**
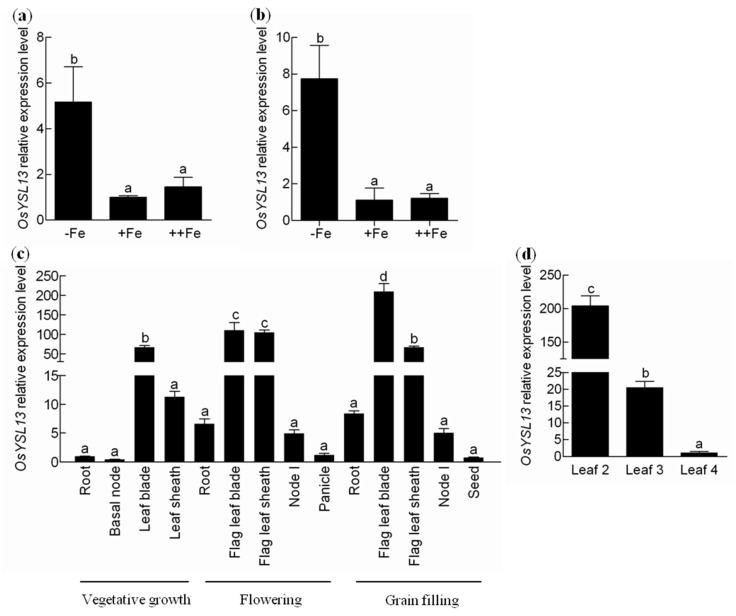
Expression analysis of *OsYSL13*. (**a**,**b**) Expression of *OsYSL13* in the roots (**a**) and shoots (**b**) under different Fe concentrations. Plants were grown for 7 days in 1/2 Kimura B nutrient solution with different Fe concentrations. 0, 2, and 20 μM FeSO_4_ were used as − Fe, + Fe, and ++ Fe treatments, respectively. The roots and shoots were collected for analysis. Relative expression level of *OsYSL13* was compared with the expression under normal nutrition condition (2 μM FeSO_4_). (**c**) Tissue-dependent expression of *OsYSL13*. Different tissues at the vegetative growth, flowering, and grain filling stages were sampled. Plants were grown in 1/2 Kimura B nutrient solution for 4 weeks and then transplanted to soil. Expression relative to the roots at the vegetative growth stage was shown. (**d**) Expression of *OsYSL13* in the different leaves. Plants were cultured in normal nutrient solution for 7 days. Leaf 4 was the youngest leaf. Different leaves (from leaves 2 to 4) were sampled for analysis. Relative expression level of *OsYSL13* was compared with the expression in leaf 4. The expression was determined by quantitative RT-PCR. *OsActin1* was used as an internal control. Data were means ± SD (*n* = 3). Means with different letters were significantly different. Analysis of variance (ANOVA) with a subsequent Duncan′s test was performed (*p* < 0.05).

It has been reported that *OsYSL13* was up-regulated by Fe deficiency in the roots [28]. In order to examine the expression pattern of *OsYSL13* in response to different Fe levels, the quantitative real-time PCR was performed. As expected, *OsYSL13* was induced by Fe deficiency both in the roots and shoots (Figure 3a,b). The expression level of *OsYSL13* under Fe excess was similar to that under normal condition (Figure 3a,b). These results suggest that *OsYSL13* is involved in Fe homeostasis. Different tissues at the vegetative growth, flowering, and grain filling stages were also sampled for the tissues-specific expression analysis of *OsYSL13*. Higher relative expression level of *OsYSL13* was found in the leaf blades and sheaths at all the three stages mentioned above, as compared with the expression of *OsYSL13* in the roots at vegetative growth (Figure 3c). Furthermore, *OsYSL13* was expressed highly in the leaf blades in comparison with the leaf sheaths at the vegetative growth and grain filling stages (Figure 3c). Among different stages, the expression of *OsYSL13* in the leaf blades was increased with the age of plants (Figure 3c). For more detailed analysis, the relative expression level of *OsYSL13* in the different leaves (from leaf 2 to leaf 4) was tested. The results showed that *OsYSL13* was more highly expressed in the older leaves than the younger leaves (Figure 3d).

### 2.2. Phenotypic Analysis of the osysl13 Mutant and OsYSL13 Overexpression Lines

**Figure 4:**
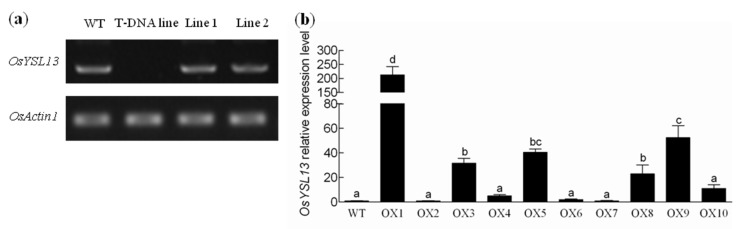
Expression identification of *OsYSL13* in the T-DNA insertion line (RMD_04Z11MA04), complementation and overexpression lines. (**a**) Expression of the full length *OsYSL13* transcript in the wild type, T-DNA insertion line (RMD_04Z11MA04) and two independent complementation lines. (**b**) Quantitative RT-PCR analysis of *OsYSL13* in the wild type and overexpression lines. Relative expression level of *OsYSL13* was compared with the expression in the wild type. Means with different letters were significantly different. ANOVA with a subsequent Duncan′s test was performed (*p* < 0.05). Data were shown as means ± SD (*n* = 3). All plant materials were grown in normal nutrient solution for 7 days. The roots were sampled for analysis. *OsActin1* was used as an internal control.

To evaluate whether *OsYSL13* was involved in Fe homeostasis, a T-DNA insertion line (RMD_04Z11MA04) of *OsYSL13* was obtained from the rice-mutant database [29]. The T-DNA was inserted into the first exon of *OsYSL13* (Figure 1a). There was no transcript of *OsYSL13* in this line, suggesting that it is a knocked-out mutant (Figure 4a). In addition, the *OsYSL13*-expressed complementation lines in the mutant background, and the *OsYSL13* overexpression lines in the wild type background were also generated. The transcript of *OsYSL13* was detected in the two independent complementation lines used in this study (Figure 4a). The expression of *OsYSL13* increased significantly among these overexpression lines obtained, except for the overexpression (OX) lines 2, 6, and 7 (Figure 4b). Among them, line 1 increased the most and line 4 increased the least (Figure 4b). Line 3 and line 5 were chosen for further research.

**Figure 5:**
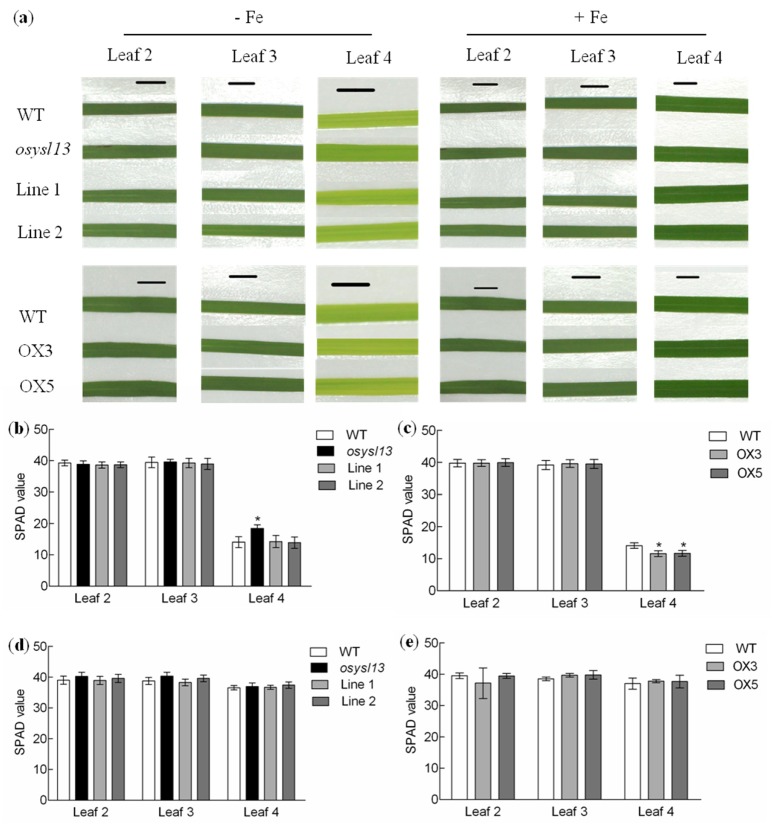
Phenotypic analysis of the *osysl13* mutant, the complementation lines (Line 1 and 2), wild type and *OsYSL13* overexpression lines (OX3 and OX5). (**a**) The fully expanded leaves of the plant materials during Fe deficiency (shown in the left) and sufficiency (shown in the right). Plants were grown in nutrient solution with or without Fe for 10 days. There were 5 leaves in each of these plant materials and the fifth leaf was not fully expanded. Scale bars = 0.5 cm. (**b**–**e**) The soil and plant analyzer development (SPAD) values of different leaves. (**b**,**d**) SPAD values of the different leaves in the *osysl13* mutant, wild-type, and complementation lines during Fe deficiency (**b**) and sufficiency (**d**). (**c**,**e**) SPAD values of different leaves in the *OsYSL13* overexpression lines and wild type during Fe deficiency (**c**) and sufficiency (**e**). The SPAD values were measured by the portable chlorophyll meter. It was difficult to measure the SPAD values of the unexpanded leaves. Significant differences compared with the wild type were determined by ANOVA (* *p* < 0.05). Data were shown as means ± SD (*n* = 3).

Plant materials, including the *osysl13* mutant, the complementation lines (Line 1 and 2), wild type and *OsYSL13* overexpression lines (OX3 and OX5) were hydroponically cultivated in nutrient solution with or without 2 μM FeSO_4_ for 10 days. There was no significant difference on the length or biomass of the roots and shoots, as compared with their corresponding wild type under both Fe deficiency and sufficiency (Figure S1). Interestingly, leaf 4 of the *osysl13* mutant, which was closest to the youngest leaf (leaf 5), was greener than that in its wild type during Fe deficiency, while the opposite result was found in the *OsYSL13* overexpression lines (Figure 5a). Moreover, the soil and plant analyzer development (SPAD) value of leaf 4 was higher in the *osysl13* mutant and lower in the *OsYSL13* overexpression lines during Fe deficiency, compared with their corresponding wild type (Figure 5b,c). Fe homeostasis may be disordered in the *osysl13* mutant and *OsYSL13* overexpression lines. The SPAD values of the other leaves under Fe deficiency and all leaves under Fe sufficiency were similar to their corresponding wild type (Figure 5b–e). SPAD values cannot reflect the Fe content quantitatively. The leaves with similar SPAD values, which were at high levels, may still have different Fe concentrations. Thus, Fe concentrations in the different leaves were analyzed.

### 2.3. Physiological Functional Analysis of OsYSL13 in Fe Homeostasis

**Figure 6:**
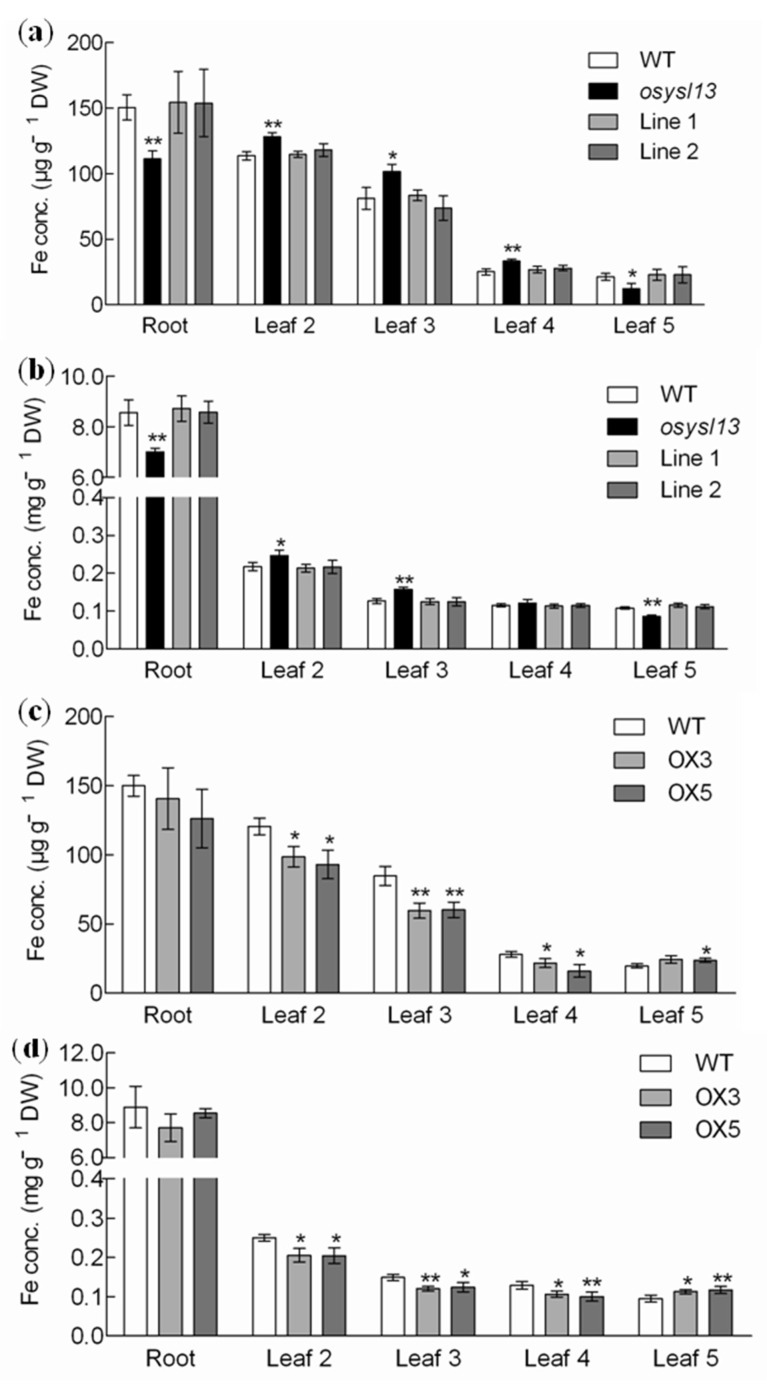
Fe concentrations analysis at the vegetative stage. (**a**,**b**) Fe concentrations in the *osysl13* mutant, wild type, and complementation lines during Fe deficiency (**a**) and sufficiency (**b**). (**c**,**d**) Fe concentrations in the *OsYSL13* overexpression lines and wild type during Fe deficiency (**c**) and sufficiency (**d**). Plants were grown in nutrient solution with or without Fe for 10 days. The roots and different leaves were sampled for analysis. Fe concentrations were determined by inductively coupled plasma mass spectrometry (ICP-MS). Significant differences compared with the wild type were determined by ANOVA (* *p* < 0.05 and ** *p* < 0.01). Data were shown as means ± SD (*n* = 3). DW, dry weight. Conc., concentration.

To gain additional insight into the function of *OsYSL13*, Fe concentrations in the roots and different leaves were determined. Higher Fe concentrations were found in the old leaves, and lower Fe concentrations were found in the youngest leaf of the *osysl13* mutant under both Fe deficiency and sufficiency, compared with its wild type and two independent complementation lines (Figure 6a,b). Opposite results were found in the *OsYSL13* overexpression lines (Figure 6c,d). These results suggest that *OsYSL13* is involved in Fe distribution to the youngest leaf. Besides, the Fe concentrations in the roots of *osysl13* mutant were reduced under both Fe deficiency and sufficiency, compared with its wild type and two independent complementation lines (Figure 6a,b). However, there was no significant difference in Fe concentrations in the roots between the *OsYSL13* overexpression lines and wild type (Figure 6c,d).

**Figure 7:**
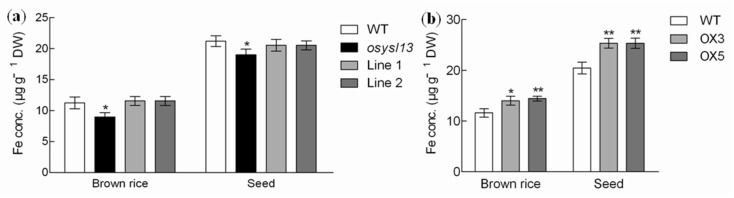
Fe concentrations analysis at the reproductive stage. (**a**) Fe concentrations in the brown rice and seeds of the *osysl13* mutant, wild-type, and complementation lines. (**b**) Fe concentrations in the brown rice and seeds of *OsYSL13* overexpression lines and wild type. Seeds were harvested from rice grown in soil. Fe concentrations were determined by ICP-MS. Significant differences compared with the wild type were determined by ANOVA (* *p* < 0.05 and ** *p* < 0.01). Data were shown as means ± SD (*n* = 3). DW, dry weight.

Many YSL members in rice and *A. thaliana* were reported to be involved in Fe distribution to the seeds [10,13,16,17,21]. To check whether *OsYSL13* plays a role in this process, Fe concentrations in the seeds and brown rice were measured. The results showed that Fe concentrations in the seeds and brown rice of *osysl13* mutant were reduced, while these were increased in the *OsYSL13* overexpression lines, compared with their corresponding wild type (Figure 7). This indicates that *OsYSL13* is also involved in Fe distribution to the seeds.

## 3. Discussion

In rice, Fe is taken up by iron-regulated transporter 1 (IRT1), natural resistance associated macrophage protein 5 (NRAMP5), and YSL15 from rhizosphere [12,17,30,31,32]. Then, it is transferred to the shoots by long distance translocation, and YSL2 is involved in this process [13,14]. YSL9 is implicated in delivering Fe into the embryo during seed development [20]. Mitochondrial iron transporter (MIT) is localized to the mitochondria and takes part in the mitochondrial Fe translocation [33]. Vacuolar iron transporter 1 (VIT1) and VIT2 are localized to the vacuolar membrane and responsible for Fe storage in vacuole [34]. YSL16 and YSL18 were also reported to be involved in Fe homeostasis, while the exact mechanisms were not clear [15,18,27]. In this study, we found that *OsYSL13* was involved in Fe distribution in rice.

### 3.1. OsYSL13 Is Localized to the Plasma Membrane

Our results show that *OsYSL13* is localized to the plasma membrane (Figure 2). Similar results were also found in most of the reported YSL members in rice and *A. thaliana*, except for YSL members in Group II [10,11,12,13,14,15,16,17,18,19,20,21,22,24,25,26,27]. *OsYSL6* has an unclear subcellular localization and takes part in Mn detoxification [24]. *AtYSL6* is localized to the chloroplast envelope and is essential for chloroplast Fe homeostasis [25]. *AtYSL4* maybe have the same subcellular localization and function as *AtYSL6* [25]. Other YSL members reported are localized to the plasma membrane and play critical roles in microelements absorption and/or translocation, while their expression pattern in response to different nutritional statuses is not all the same [10,11,12,13,14,15,16,17,18,19,20,21,22,24,25,26,27].

### 3.2. OsYSL13 Is Involved in Fe Distribution to the Youngest Leaf

*AtYSL1-3* are reduced during Fe deficiency, and *AtYSL4*, *6* are induced by Fe excess [11,16,19,21,25]. Unlike the reported YSL members in *A. thaliana*, *OsYSL2*, *9*, *15*, and *16* are induced by Fe deficiency [12,13,14,15,17,18,20,22,24]. In this study, *OsYSL13* was also found to be induced during Fe deficiency (Figure 3a,b). YSL members in rice and *A. thaliana* may play different roles in response to Fe starvation. Moreover, *OsYSL13* was highly expressed in the leaves, especially in the leaf blades (Figure 3c). Higher expression level of *OsYSL13* was found in the older leaves than in the younger leaves (Figure 3d), which was similar with the expression of *OsYSL16* (Figure S2). It was reported that *OsYSL16* was involved in Cu distribution among the younger leaves [22]. Higher concentrations of Cu were found in the older leaves, and lower concentrations of Cu were found in the younger leaves of the *osysl16* mutant compared with the wild type [22]. Disruption of *OsYSL13* resulted in increased Fe concentrations in the old leaves and reduced Fe concentrations in the youngest leaf during both Fe deficiency and sufficiency (Figure 6a,b). In addition, overexpression of *OsYSL13* led to opposite results (Figure 6c,d). This indicates that *OsYSL13* is involved in Fe distribution to the youngest leaf.

### 3.3. OsYSL13 May Be Involved in Fe Accumulation in the Roots

Same transporter expressed in the different tissues may also take part in different translocation process. *OsYSL16* was highly expressed in the leaves and node I, and it was involved in Cu distribution to the younger leaves and panicles [22]. Besides, *OsYSL16* was also highly expressed in the rachilla, palea, and lemma, and it was essential for Cu distribution to the stamens [23]. It was reported that *OsFRDL1* was expressed in the roots and node I, and it was responsible for Fe root-to-shoot translocation and Fe distribution to the reproductive organs [35,36]. *OsYSL13* was not only expressed in the leaves, it was also expressed in the roots (Figure 3c). Lower Fe concentrations in the roots were found in the *osysl13* mutant under normal nutrition and Fe deficiency, compared with the wild-type and two independent complementation lines (Figure 6a,b). *OsYSL13* may be also involved in Fe accumulation in the roots. However, Fe concentrations were not increased in the roots of *OsYSL13* overexpression lines (Figure 6c,d). Opposite results were not always found between the mutant and overexpression lines. For example, Fe concentrations in the seeds and shoots of *OsYSL2i* and *OsYSL2* overexpression lines were both lower, while the Fe concentrations were both higher in the roots [13]. Some unknown mechanisms may be involved.

### 3.4. OsYSL13 Is Involved in Fe Distribution to the Seeds

Rice is one of the major food crops in the world. It is important to improve the Fe concentrations in rice, especially for the grains. However, Fe concentrations in the grains ranged slightly under different Fe levels in soil and culture solution [37]. Identification of the transporters involved in Fe distribution to the seeds will be helpful to enhance Fe concentrations in the grains by transgenic technology. It has been reported that *OsYSL2*, *OsYSL15*, *AtYSL1,* and *AtYSL3* are all essential for Fe distribution to the seeds [10,13,16,17,21]. The Fe concentrations were decreased in the seeds of *OsYSL2i*, *atysl1* mutant, *atysl1atysl3* double mutant, and *OsYSL2* overexpression lines driven by the CaMV35S promoter [10,13,16,21]. Besides, higher Fe concentrations were found in the seeds of *OsYSL15* overexpression lines [17]. Moreover, the polished rice with a high Fe concentration was produced by overexpression of *OsYSL2* driven by the rice sucrose transporter 1 (*OsSUT1*) promoter [13]. *OsYSL9* is responsible for Fe translocation from the endosperm to embryo [20]. In this study, lower Fe concentrations were found in the seeds and brown rice of the *osysl13* mutant, while higher Fe concentrations were found in the *OsYSL13* overexpression lines (Figure 7). Like these YSL members mentioned above, *OsYSL13* is also involved in Fe distribution to the seeds.

## 4. Materials and Methods

### 4.1. Plant Materials

A T-DNA insertion line (RMD_04Z11MA04) of *OsYSL13*, its wild-type rice (*Oryza sativa* cv. Zhonghua 11), and two transgenic complementation lines were used in this study. In addition, *OsYSL13* overexpression lines were also produced. The T-DNA insertion line was acquired from the rice mutant database [29]. Primers used for T-DNA insertion line genotyping and the generation of transgenic lines are shown as Table S1. The sequence of complementation includes a ~2.5 kb promoter region, coding region, and ~0.5 kb downstream region. The full length of *OsYSL13* cDNA sequence was also amplified for the overexpression. pDONR was used as the entry vectors. pEarleyGate 301 and pGWB2 were chosen as final recombination for the complementation and the overexpression of *OsYSL13*, respectively [38,39]. These fragments were first recombined into pDONR and finally recombined into pEarleyGate 301 and pGWB2, respectively. The resultant plasmid was transferred to *Agrobacterium tumefaciens* (strain EHA105). Transformation to callus (the T-DNA insertion mutant for complementation and *Oryza sativa* cv. *Nipponbare* for overexpression) was carried out as described previously [40]. More than 10 lines were obtained, and two of them were chosen for further research.

### 4.2. Growth Conditions

Seeds were soaked in water for three days at 30 °C in darkness and then transferred to a net floating on 0.5 mM CaCl_2_ solution. On the day 8, CaCl_2_ solution was replaced by one-half-strength Kimura B solution (pH 5.5). On the day 11, the seedlings were transferred to a 1.2-L plastic pot containing the nutrient solution with different treatments. All seedlings were grown in a greenhouse at 28 °C to 32 °C. The nutrient was renewed every 2 days. The nutrient solution used contained following macronutrients: (mM), (NH_4_)_2_SO_4_ (0.18), MgSO_4_·7H_2_O (0.27), KNO_3_ (0.09), Ca(NO_3_)_2_·4H_2_O (0.18), and KH_2_PO_4_ (0.09); and micronutrients (μM), MnCl_2_·4H_2_O (0.5), H_3_BO_3_ (3), (NH_4_)_6_Mo_7_O_2_·4H_2_O (1), ZnSO_4_·7H_2_O (0.4), FeSO_4_·7H_2_O (2 or free), and CuSO_4_·5H_2_O (0.2). After four weeks of growth, the seedlings were transplanted to a 15-L plastic pot filled with rice paddy soils in a greenhouse at Nanjing Agricultural University (Nanjing, China).

### 4.3. Gene Expression Analysis

To check the expression pattern of *OsYSL13* in response to different Fe conditions, 10-day-old seedlings were exposed to solution with different Fe concentrations (0, 2, 20 μM FeSO_4_) for 1 week. Then, the seedlings were collected as the roots and shoots. For the tissue-specific expression analysis, 3-, 14-, and 16-week-old plants were prepared to reveal the expression level of *OsYSL13* during the vegetative growth, flowering, and grain filling stages, respectively. Different tissues, including root, basal node, leaf blade, leaf sheath, node I, panicle, and seed, were sampled at these stages accordingly. Moreover, different leaves at the vegetative growth stage were also sampled for a detailed expression pattern analysis of *OsYSL13*. Total RNA was extracted by using TaKaRaMiniBEST Universal RNA Extraction Kit (TaKaRa, Dalian, China) and then converted to cDNA by using TaKaRaPrimeScript™ 1st Strand cDNA Synthesis Kit (TaKaRa, Dalian, China). The expression of *OsYSL13* was measured by quantitative real-time PCR, and the primers used are listed in Table S1. Mastercycler ep realplex (Eppendorf, Hamburg, Germany) was used for quantitative real-time PCR. Specific cDNAs were amplified by SYBR^®^ Premix Ex Taq^TM^ (TaKaRa, Dalian, China). *OsActin1* (LOC_Os03g50885) was used as an internal control. ΔΔ*C*t (cycle threshold) method was used to calculate the relative expression of *OsYSL13*. Three biological replicates were taken for performing the experiment.

### 4.4. Subcellular Localization

To investigate the subcellular localization of *OsYSL13*, full-length cDNA fragment of *OsYSL13* was amplified by PCR from the rice cDNA library, using the primers provided in Table S1. To generate sGFP (synthetic Green Fluorescent Protein)-OsYSL13 fusion protein, the fragment was cloned into pBluescript SK vector behind GFP. The termination codon of GFP was removed. The onion epidermal cells and rice protoplasts were used for the transient expression of the resultant plasmid and 35S-sGFP control vector as previously reported [22,41]. Finally, GFP fluorescence signal was observed with laser confocal microscope (UltraVIEW VOX, PerkinElmer, Waltham, MA, USA).

### 4.5. Measurement of SPAD Values

The portable chlorophyll meter (SPAD-502; Konica Minolta Sensing, Osaka, Japan) was used to measure the SPAD values of the fully expanded leaves, which can reflect the relative content of chlorophyll. Three biological replicates were used.

### 4.6. Determination of Fe Concentrations

Plant materials were grown in nutrient solution with or without 2 μM FeSO_4_ for 10 days. Samples were washed with 5 mM CaCl_2_ three times. After that, the roots and different leaves were harvested. Besides, the brown rice and seeds from plant materials grown in soil were also collected for the determination of Fe concentrations. A mixture of concentrated HNO_3_ and HClO_4_ (87:13, (*v*/*v*)) was used for plants digestion according to Zhao et al. [42]. The Fe concentrations were measured by ICP-MS. Three biological replicates were used.

### 4.7. Statistical Analysis

SPSS statistical software (17.0, SPSS Inc., Chicago, IL, USA) was used for statistical analysis. Data were exhibited as means ± SD (*n* = 3). Significant differences were determined by ANOVA (* *p* < 0.05, ** *p* < 0.01, and *** *p* < 0.001).

### 4.8. Accession Number

Sequence data from this article can be found in the GenBank/EMBL databases under accession number Os04g0524500 for *OsYSL13*.

## 5. Conclusions

The youngest leaves are the developing leaves which are not fully expanded. Microelements translocation driven by the leaf transpiration cannot satisfy the nutritional requirements for the youngest leaves. Our results showed that *OsYSL13* was involved in Fe distribution from the old leaves to the youngest leaves and seeds. As a novel transporter required for Fe distribution to the seeds, *OsYSL13* can be used as a candidate target for iron biofortification in rice.

## Supplementary Materials

Supplementary materials can be found at http://www.mdpi.com/1422-0067/19/11/3537/s1.

## Abbreviations

ANOVA	analysis of variance
DMA	deoxymugineic acid
ICP-MS	inductively coupled plasma mass spectrometry
IRT	iron-regulated transporter
MIT	mitochondrial iron transporter
MNM	micronutrient malnutrition
NA	nicotianamine
NRAMP	natural resistance associated macrophage protein
OX	overexpression
SPAD	soil and plant analyzer development
sGFP	synthetic green fluorescent protein
SUT	sucrose transporter
VIT	vacuolar iron transporter
YSL	yellow stripe-like
