# Supplementary material for: OsYSL13 Is Involved in Iron Distribution in Rice
Source: Int J Mol Sci. 2018 Nov 9;19(11):3537. doi: 10.3390/ijms19113537 (PMC6274735; doi:10.3390/ijms19113537)
Supplement: Supplementary file 1 [file ijms-19-03537-s001.pdf]

International Journal of
Molecular Sciences

MDPI

Supplementary Materials:

Table S1. Primers used in this paper.

Purpose	Name	Sequences
T-DNA insertion line genotyping	T U	GACGTGGAGATGGTGGAGG
	T L	CAGCAGCGAAATTGATGGA
	NL	AATCCAGATCCCCCGAATTA
Complementation Test	C U	GGGGACAAGTTTGTACAAAAAAGCAGGCTTGCC AGTTGCAAGTATTGATC
	C L	GGGGACCACTTTGTACAAGAAAGCTGGGTCTCA AGCATATGTCTTCTAC
Generation of *OsYSL13* overexpression lines	OX U	GGGGACAAGTTTGTACAAAAAAGCAGGCTATGG CGACCGTTCCTACGCC
	OX L	GGGGACCACTTTGTACAAGAAAGCTGGGTTTATT TTCCCAATGTTGTAA
mRNA expression identification of T-DNA insertion line	I U	ATGGCGACCGTTCCTACGCC
	I L	TTATTTTCCCAATGTTGTAA
Quantitative real-time PCR	Q 13 U	CGGGTCTTCATAGCCATTGCC
	Q 13 L	CGCTCCTCGTCGTATGAGACA
	Q 16 U	TTGCTCCCTCTCCGAAAGGTG
	Q 16 L	GCGTGTGAAACCCGTTTATGA
	Q A U	AGACCTTCAACACCCCTGCT
	Q A L	GTCCCTCACAATTTCCCGCT
Subcellular localization	S U	CACTCACGGCATGGACGAGCTGTACAAGATGGC GACCGTTCCTACGCC
	S L	TGAACGATCTGCAGCCGGGCGGCCGCTTATTTTC CCAATGTTGTAA

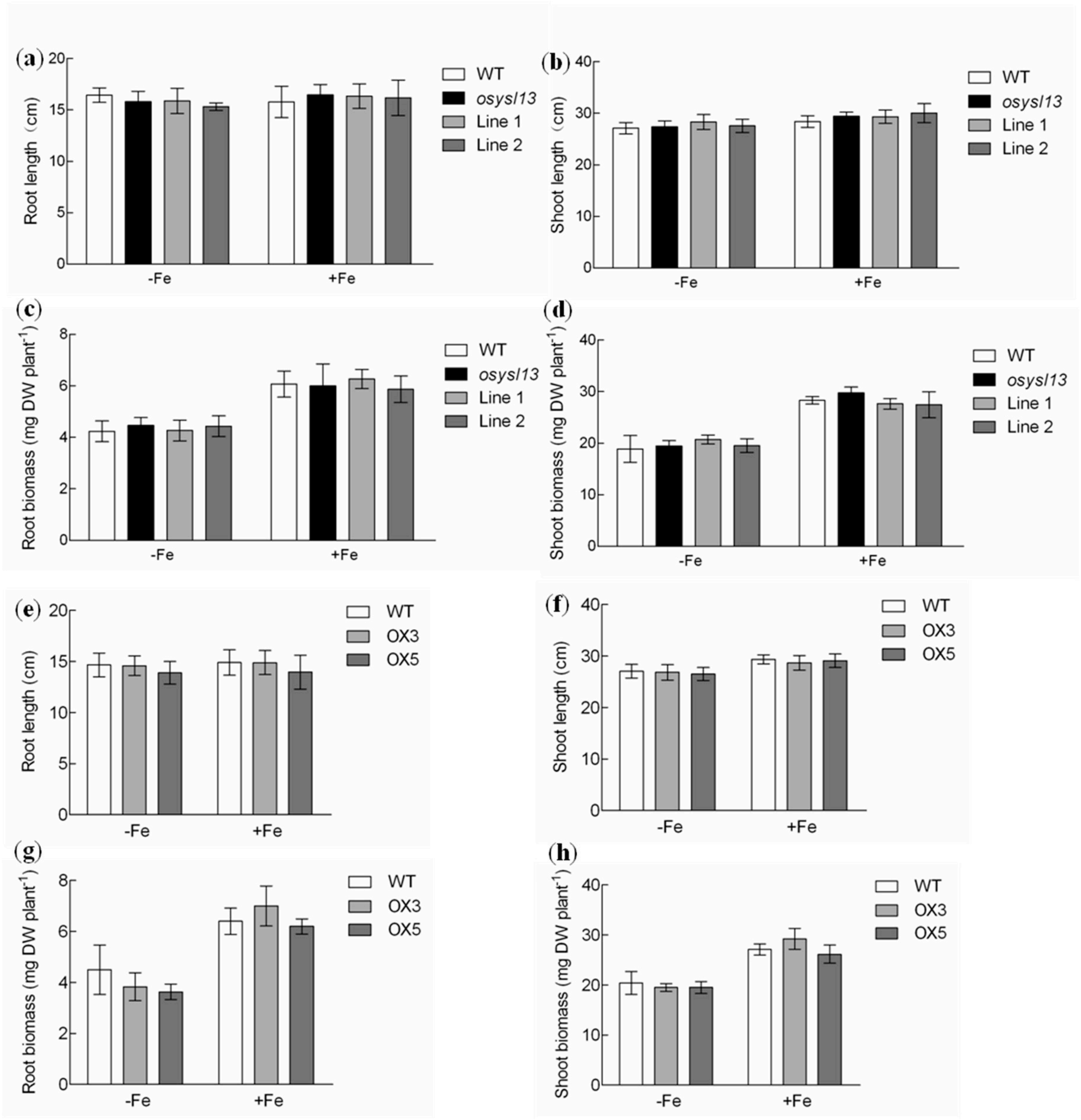

Figures S1. Plants growth during Fe deficiency and sufficiency. (**a**-**d**) The length of the roots (**a**) and shoots (**b**), and the biomass of the roots (**c**) and shoots (**d**) in the *osysl13* mutant, wild type and complementation lines. (**e**-**h**) The length of the roots (**e**) and shoots (**f**), and the biomass of the roots (**g**) and shoots (**h**) in the *OsYSL13* overexpression lines and wild type. Plant materials, including *osysl13* mutant, *OsYSL13* overexpression lines, their corresponding wild type and complementation lines, were grown in nutrient solution with or without Fe for 10 days. Significant differences compared with the wild type were determined by ANOVA ($P<0.05$). Data were shown as means ± SD ($n=3$). DW, dry weight.

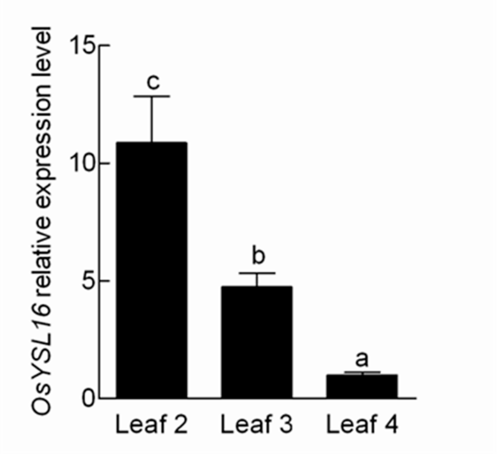

Figures S2. Expression of *OsYSL16* in different leaves. Plants were cultured in normal nutrient solution for 7 days. The leaf 4 was the newest unfully expanded leaf. Different leaves (from leaf 2 to 4) were sampled for analysis. Relative expression level of *OsYSL16* was compared with the expression in the leaf 4. The expression was determined by quantitative RT-PCR. *OsActin1* was used as an internal control. Data were means ± SD (n=3). Means with different letters were significantly different. ANOVA with a subsequent Duncan's test was performed ($P<0.05$).
